# A Survey of Occupational Therapists on a New Tool for Sensory Processing

**DOI:** 10.1155/2020/5909347

**Published:** 2020-02-28

**Authors:** Caroline J. Mills, Elisabeth Michail, Rosalind A. Bye

**Affiliations:** Occupational Therapy, School of Health Sciences, Western Sydney University, NSW, Australia

## Abstract

Occupational therapy is the leading profession with regard to supporting children who experience difficulties with occupations as a result of sensory processing differences. However, there are mixed reports with regard to the efficacy of various sensory interventions and approaches, leaving little clear guidance for occupational therapists supporting children with these difficulties. The Sensory Form is a planning tool developed in 2017 to guide occupational therapists in their professional reasoning for assessment and intervention of sensory processing differences. To date, no research has been conducted on its use. Researchers introduced the tool to 20 occupational therapists with relevant experience and conducted an online survey of their perceptions about The Sensory Form. Findings were analysed using descriptive statistics and qualitative content analysis. Therapists reported that they found the tool acceptable for use, described key strengths and weaknesses of The Sensory Form, and outlined changes to improve the tool. The Sensory Form may have an application in guiding the practice of therapists supporting children with sensory processing differences. Further development of associated resources may be warranted.

## 1. Introduction

Sensory processing involves the intake and processing of information through the senses and is unique to individuals [1]. When sensory processing is atypical, it can have a negative impact on participation in daily life and occupations including self-care [2], social participation [3], academic performance [4], and adaptive skills [5]. Sensory processing differences are often observed in children with clinical diagnoses such as autism and attention deficit hyperactivity disorder [6] and in adults with mental illness [7].

Occupational therapists are considered leaders in assessment and intervention for sensory processing differences in various populations, particularly children [7, 8]. A number of standardised assessments exist for the purpose of evaluating sensory processing, for example, The Sensory Profile 2 (SP2) [9], The Adolescent Adult Sensory Profile (AASP) [10], and The Sensory Processing Measure (SPM) [11]. While these assessments are widely used, there are limitations in terms of how therapists link assessed sensory processing differences to the child's participation in daily occupations and how understanding the relationship between sensory processing and participation then guides the selection of appropriate interventions to enhance participation [12].

A number of intervention approaches are adopted by occupational therapists in addressing sensory processing differences in children. First, Ayres' Sensory Integration Therapy (SIT) is a remedial clinic-based approach providing enhanced sensation in the context of a challenging task, with the aim of eliciting an adaptive response from the child [13, 14]. SIT studies have yielded mixed results. Schoen and colleagues [14] concluded that SIT is an evidence-based practice for children with autism, while other studies have concluded that SIT is not a suitable intervention for children with developmental disabilities [15]. Issues around fidelity and study design may account for different results obtained in SIT outcome studies [5]. Second, sensory-based interventions, also termed “sensory-specific techniques,” have been reported in the literature and consist of a range of interventions which occur outside of a clinic-based setting. These include interventions such as weighted vests, slow linear swinging, and therapeutic listening, which have limited evidence of efficacy reported [16]. Emerging evidence has been observed for the use of a Sensory Activity Schedule (SAS) in a school context [17, 18]. The SAS is described as comprising accommodation and sensory self-regulation, assisting children to manage sensory challenges in context, rather than “fixing” children's sensory processing. Third, modifications to the sensory environment have good evidence in relation to adopting universal design principles [19], and modifying lighting and sound absorption [20]. Ashburner et al. [19] presented a clinical framework for supporting participation by those on the autism spectrum who present with sensory processing differences. The framework offers universal and individual strategies for supporting sensory differences. Ashburner et al. describe accommodations and adaptive strategies such as noise cancelling headphones, targeted sensory input in the form of movement breaks, and behavioural strategies. Overall, however, there is a lack of clarity in intervention definitions and mixed reports of efficacy across various populations [21], leaving little to guide therapists in their practice to support children's sensory processing differences in a way that maximises participation/performance in daily life activities.

Assessment and intervention planning are key stages of the occupational therapy problem-solving process [22] which, if done well, can ensure that a child's needs and goals are identified and met. Professional reasoning during these stages needs to ensure therapists are attuned to particular aspects of a child's performance that may be impacting on participation in various settings. One challenge is that the professional reasoning of novice health professionals can be limited in identifying or hypothesising the causes of clients' challenges, potentially leading to the design of an intervention plan that does not address the difficulties at hand and therefore does not meet a client's needs [23–25]. The use of tools such as checklists, assessment forms, models, or frameworks and the like can encourage a more comprehensive and systematic approach to professional reasoning, ensuring that there are structured prompts to consider all the factors that might be at play in a given situation [26, 27].

There are few published studies other than Ashburner et al. [19] offering practical professional reasoning guidance to therapists [27]. No other studies were located that guide professional reasoning in relation to sensory processing differences, with no research specifically regarding how a more structured approach, such as a tool or checklist to guide reasoning around sensory processing differences and participation, might impact a therapist's planning.

**Figure 1:**
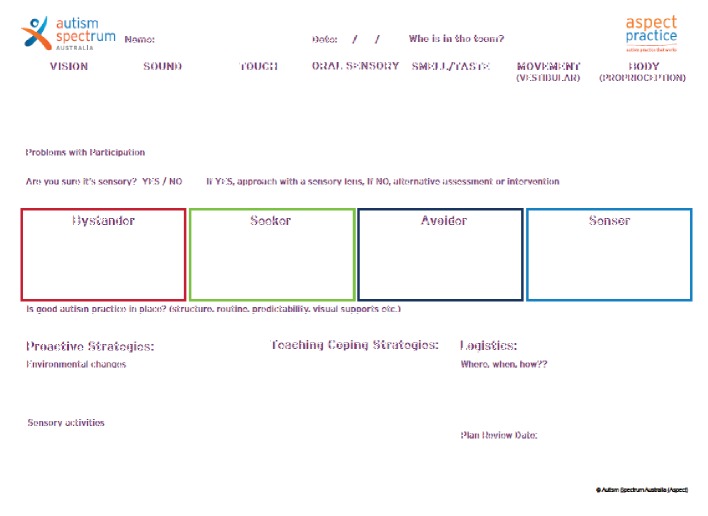
The Sensory Form.

The tool that is the focus of the present study, titled The Sensory Form (Figure 1), was developed by a large not-for-profit organisation in Australia for use with people on the autism spectrum. The tool was developed by the first author, an experienced occupational therapist, in collaboration with an autism professional with a psychology background, and has been in use since 2017. The Sensory Form has been presented at conferences in Australia and internationally and was freely available to access online at the time of this research. The first author was employed by the organisation at the time of development of The Sensory Form, but not at the time of the research. The organisation that designed The Sensory Form gave written permission for researchers to conduct research on the form.

Given the paucity of tools to guide professional reasoning in assessment and intervention for all people with sensory processing differences, the researchers aimed to explore the utility of The Sensory Form for children with sensory challenges. In addition, some people with sensory processing differences may not encounter an occupational therapist, but instead be seen by other health professionals, or educational professionals such as teachers, or be supported by their parents. Therefore, it was also important to explore whether occupational therapists considered that The Sensory Form was a tool that could be utilised by nonoccupational therapists, if given training, who encounter people with sensory processing differences. Researchers wanted to explore whether therapists felt the tool could prompt consideration of how sensory processing might be impacting performance and participation, and the kinds of strategies that could be implemented to enhance participation in context.

The aim of the present study was therefore to determine the perceptions of occupational therapists, with experience in sensory processing across a wide range of client groups, to determine their perceptions of the suitability of The Sensory Form when working with children with sensory processing differences. The key research questions were (i) what are the perceptions of occupational therapists regarding The Sensory Form as a tool for assessment and intervention planning for children with sensory processing differences and (ii) could The Sensory Form be utilised by nonoccupational therapists, such as allied health professionals, teachers, and parents, if given training, to identify and address sensory processing differences in children?

## 2. Materials and Methods

### 2.1. Research Design

This study utilised a descriptive survey methodology to design and implement a brief online survey containing both open-ended and Likert responses to gather therapists' perspectives regarding The Sensory Form. Descriptive survey methodology allows researchers to collect data on respondents' perspectives and at the same time, collect detailed information pertaining to respondents' demographic profiles [28, 29]. Furthermore, this approach allows researchers to ask a specific set of questions in the exact same way to all respondents [30]. The online delivery has the added benefit of enabling the involvement of respondents from diverse locations.

Ethical approval was obtained from Western Sydney University's Human Ethics Committee, approval number H12874. Therapists gave informed written consent for their participation.

### 2.2. Recruitment and Respondents

Respondents were 20 occupational therapists who had experience working with children with sensory processing differences. Purposive and snowball sampling was used to recruit occupational therapists with specific knowledge on the topic of sensory processing differences. Purposeful selection of respondents can be used to highlight a particular perspective important to the research aim [31]. Occupational therapists were recruited from professional networks, including closed social media groups with a focus on sensory processing, private practices, the local health district, and universities. Therapists were also asked to recruit other suitable participants in their networks. To prevent bias, no occupational therapists were recruited from the organisation which developed The Sensory Form.

Table 1 describes therapist characteristics. Descriptive statistics were used to organise and summarise demographic information obtained from therapists to create a respondent profile [32]. Therapists had an average of 14.48 years of occupational therapy practice experience and were asked to identify relevant postgraduate training they had received. Fourteen therapists reported they had completed various types of additional training, including Wilbarger's brushing protocol, therapeutic listening, DIR (Developmental, Individual Difference, Relationship based) Floortime, Alert, and Sensory Integration Training, as well as workplace continuing professional development. In presenting details about their additional training, therapists also identified well-known occupational therapy scholars in the field of sensory processing, whose work they had read, or whose training they had attended, including Winnie Dunn, Theresa May-Benson, Christine Chapparo, Sheila Frick, and Tina Champagne.

### 2.3. Research Process

**Figure 2:**
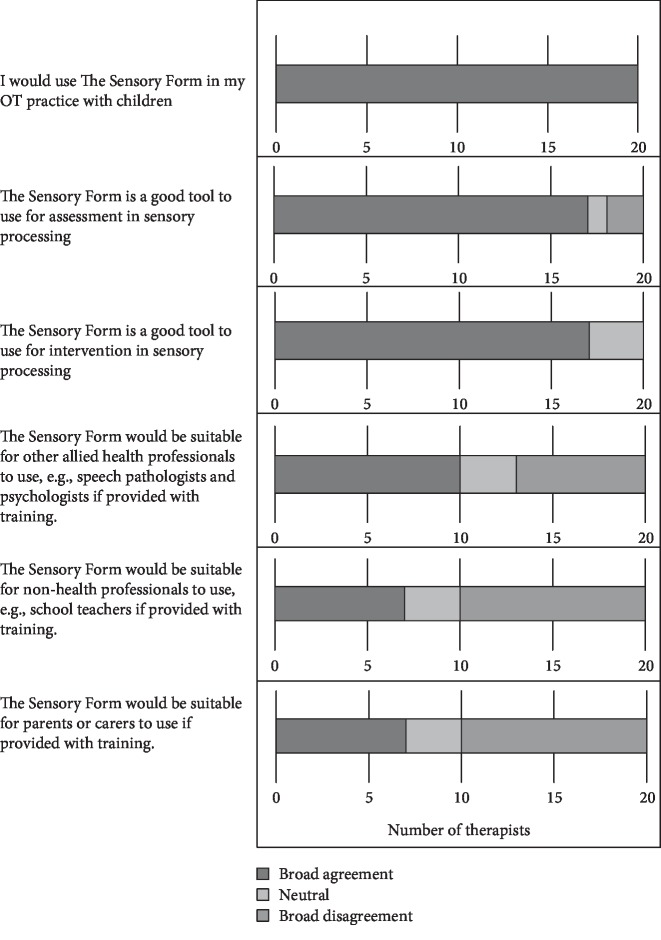
Likert scale responses from occupational therapists.

Following recruitment, therapists were invited to view a 25-minute webinar which reviewed sensory processing, explained The Sensory Form in detail, and provided a case study application of The Sensory Form with a child on the autism spectrum who had sensory processing differences causing difficulties in her home and school setting. This case study was developed based on the first author's practice experience with children and demonstrated the use of The Sensory Form with a real child. Handout materials from this webinar are available as Supplementary File One ([Supplementary-material supplementary-material-1]). Following the webinar, therapists were given a web link to access The Sensory Form online. The Sensory Form given to participants following the webinar is presented as Figure 1. Therapists were asked to review the Form in their own time after the webinar and complete a short, online survey provided through the Qualtrics platform. The survey used open-ended questions to capture participant perceptions of the strengths and weaknesses of The Sensory Form and also asked therapists to suggest changes which could be made to The Sensory Form to enhance usability. Therapists were also asked the extent to which they agreed with statements about The Sensory Form on a five-point Likert scale. These statements addressed the form's suitability for use by occupational therapists and, if provided with training, allied health professionals or parents of children with sensory processing differences. Therapists rated the extent to which they agreed with various statements about The Sensory Form. Likert scale statements are presented in Figure 2.

### 2.4. The Tool: The Sensory Form

The Sensory Form is a one-page tool (see Figure 1), consisting of eight sections to guide the user through a process of assessment and intervention planning for a person with sensory processing differences. Initial sections of the form relate to sensory processing assessment. The first section consists of boxes in which users describe observed sensory behaviours in relation to the senses, namely, vision, sound, touch, oral sensory, smell/taste, vestibular, and proprioception processing. The second section, titled problems with participation, prompts the user to reflect on whether the observations of sensory processing in the boxes above impact on a person's participation in their daily activities and occupations. This is important as not all observed sensory behaviours pose a problem for participation [19] and therefore may not need to be addressed with intervention [33]. Following this, in the third section, users are prompted with the question, “are you sure it's sensory?” This prompt serves to prevent the user from attributing all presenting problems as sensory problems. This section encourages users to take a functional behaviour analysis approach and consider the purpose or “function” behind a child's behaviour [34], as observed problems with participation in children can result from a complex interplay of emotional, social, cognitive, or other nonsensory factors [19]. The fourth section of the form presents the four categories of sensory processing as presented in Dunn's Sensory Processing Framework, namely, Bystander, Seeker, Avoider, and Sensor [9]. Dunn's framework proposed that four sensory styles can be observed based on the point at which a person registers sensory input (termed neurological threshold) and how they respond behaviourally to the sensory input (passive or active behavioural responses can be observed). Children are often observed to have more than one sensory style [12]. The Sensory Form contains a fifth section which asked the question, “Is good autism practice in place?” This reflects that the organisation that initially developed The Sensory Form has a particular focus on supporting people on the autism spectrum. This section is included to capture evidence-based supports which may not be sensory in nature, but which can have an impact on how a child responds to and utilises sensory information in context. For children on the autism spectrum, “good autism practice” might include use of visual supports [35], augmentative and alternative communication strategies [36], and autism friendly environments [37] tailored to the individual needs of each person [35].

The bottom section of the form consists of three subsections, comprising different activities and considerations for intervention planning to address the sensory processing differences and the negative impact they may have on participation as identified by The Sensory Form. In line with Dunn, Cox, Foster, Mische-Lawson, and Tanquary's [38] approach, proactive and coping strategies are not utilised to remediate sensory processing differences, but rather to be integrated into a person's context to enable their participation. The sixth section of the form comprises proactive strategies related to environmental changes and sensory activities. Environmental modifications including reducing distractions, considering sound and lighting, and considering the physical layout of the environment are examples of strategies that may be indicated here [18, 20]. Examples of sensory activities that can be used and are relevant to this section include fidget items, movement breaks, and activities involving heavy work and deep pressure which can increase engagement in classroom tasks [18], or to reinforce participation [39]. The seventh section of The Sensory Form prompts users to consider specific coping skills that a person with sensory processing differences may want to or be able to learn, for example, asking for a break or learning to move away from distressing sensory input. Specific strategies can assist with regulating physiological arousal levels in different environments [19], and enhancing participation [40]. The eighth box, titled “Logistics,” comprises practical considerations for how supports will be implemented considering individual circumstances and context, such as available and required resources and personnel support, and how outcomes will be measured [41]. The Form then prompts for a plan review date, acknowledging that this is an important step in occupational therapy service provision [22].

### 2.5. Data Analysis

Data were analysed in the following ways. For ease of presentation, Likert scale responses were grouped into “broad agreement” (comprising strongly agree and agree), neutral, and “broad disagreement” (comprising strongly disagree and disagree) and presented in Figure 2.

Open-ended responses relating to the strengths, weaknesses, and suggested changes to The Sensory Form were analysed qualitatively. Written survey responses were collated in the Qualtrics survey program and responses were converted into a word document. The constant comparative approach of open coding of qualitative data was used to group like data into categories pertaining to strengths, weaknesses, and suggested changes [42, 43]. Responses to each open-ended question were coded separately by the first and last authors by comparing and contrasting responses under each question, labelling with codes and developing tentative categories [42, 43]. Both authors then met to discuss and reach agreement on final coding and categorical groupings and titles.

## 3. Results

### 3.1. Likert Scale Responses

As shown in Figure 2, all therapists demonstrated broad agreement with the statement that they would use The Sensory Form in their practice with children. Seventeen therapists expressed broad agreement that The Sensory Form was suitable for assessment and intervention. Two therapists disagreed with the statement that The Sensory Form was a suitable tool for assessment.

With regard to the suitability of a nonoccupational therapist using The Sensory Form, responses were mixed. Almost half of the therapists (9) were in broad agreement that the tool was suitable for use by other health professionals and seven therapists were in broad disagreement. When asked if The Sensory Form was suitable for use by teachers or parents, half of the therapists broadly disagreed (10), with seven agreeing.

### 3.2. Strengths of The Sensory Form

Four categories emerged describing the strengths of The Sensory Form. These categories are outlined in Table 2 with illustrative quotes. The first strength category, *participation focus*, identified that a key strength of The Sensory Form was that it prompted therapists to move from observing sensory processing, to then thinking about how that sensory processing might impact on an individual's participation. The second strength category, *facilitates professional reasoning links between assessment and intervention*, revealed that therapists believed that The Sensory Form provided an opportunity for reflection about observations and assessments and how reflecting on these results could positively impact intervention planning. The structured nature of The Sensory Form enabled therapists to engage in professional reasoning that identified goals and interventions, linking these aspects of the problem-solving process to the assessment of sensory processing. The third strength category, *encourages collaboration with others*, highlighted therapists views that The Sensory Form also played a role in prompting therapists to consider others in the team, including parents, teachers, and other health professionals, as well as providing a clear and concise summary of findings pertaining to sensory processing and participation that could easily be shared with others. The fourth strength category, *simple and easy to use*, summarised therapists' positive comments that The Sensory Form was clear and logically ordered, which aided their reasoning, and included complex information in one place.

### 3.3. Weaknesses of The Sensory Form

Three categories emerged in relation to weaknesses of The Sensory Form, and these are outlined in Table 3 with direct quotes from therapists. The first weakness category, *requires OT background knowledge, experience, and training*, highlights therapists' concerns regarding the usefulness and appropriateness of The Sensory Form use by those without specific knowledge and training in sensory processing. This may limit the utility of The Sensory Form in situations where there is no occupational therapist present. Therapists did comment that there was merit in having a form more suitable to nontherapists, with one possible option being the development of a Sensory Form that was specifically designed for parents. The second weakness category, *supports reasoning in a limited way*, was related to professional reasoning and whether The Sensory Form would provide a thorough enough assessment to guide intervention choices. Comments primarily indicated that the form had utility to facilitate reasoning, but there was a limit to its utility, with several therapists commenting that other assessment tools or outcome measures would be required if a therapist was to undertake a more detailed approach to client management. The third weakness category, *expand for clarity and logic*, is related to the formatting of The Sensory Form, with a number of therapists reporting issues with layout and limited available space for writing on the form. Suggestions included expanding the size of the space for comments and ordering content so reasoning logic was made clear.

### 3.4. Recommended Changes to The Sensory Form

Four recommendations emerged in relation to suggested changes to The Sensory Form. First, therapists suggested changes to how the senses were presented and assessed, to enable more specificity and clarity, including the addition of the additional sensory area, interoception, to the top section of the form. Interoception consists of the perception of one's internal body state, including, pain, emotion, and health [44]. Therapist 10 said “try to incorporate a clearer depiction of the sensory responses.” Second, therapists recommended that the “Problems with Participation” section be made larger and suggested that “time of day (arousal) and duration of activities [could be] reflected in the form” (T 11). Third, therapists recommended that the presentation of Dunn's Sensory Processing Framework in The Sensory Form be reorganised to make this information clearer for the user. Therapists recognised the complexity in Dunn's sensory styles, in that children may be a Bystander in one sensory style, but a Seeker in another. Therapist 18 stated “I would remove the boxes of bystander, seeker, avoider, sensitivity and prompt for that info to be put under each sense.” In addition, Therapist 20 recommended the terms “passive and active [could be] added to the corresponding [sensory] processing pattern.” Therapist 2 suggested a “picture from [the] Winnie Dunn model … to aid understanding by others.” Finally, recommendations were made regarding additional prompts for users to enhance professional reasoning and logic. “At teaching coping strategies: Add examples to choose from.” (T 17).

## 4. Discussion

This study was aimed at understanding the perceptions of 20 occupational therapists in order to answer the key research questions: (i) what are the perceptions of occupational therapists regarding The Sensory Form as a tool for assessment and intervention planning for children with sensory processing differences and (ii) could The Sensory Form could be utilised by nonoccupational therapists, if given training, such as allied health professionals, teachers, and parents, to identify and address sensory processing challenges in children? A number of key findings were obtained.

First, therapists had positive perceptions about The Sensory Form's utility in guiding their practice around appropriate assessment and intervention to address sensory processing differences. All therapists were in broad agreement that they would use The Sensory Form in their occupational therapy practice with children. A key strength of The Sensory Form that emerged was the “problems with participation” section; it was indicated that this section may allow therapists to remain occupation focused, that is, to interpret sensory observations in terms of activity and participation. This is important in an area of practice where remediating underlying deficits is reported to have limited evidence [45]. The Sensory Form does not promote interventions that remediate sensory processing differences, but rather is aimed at facilitating occupational therapists' selection of appropriate accommodation and self-regulation interventions which can target participation in daily contexts.

The Sensory Form was regarded by therapists as a tool that encouraged reflection on aspects of professional reasoning, by prompting therapists to link stages of the problem-solving process during use of The Sensory Form. This outcome is beneficial to therapists and particularly novice clinicians who may require a more structured approach to improving their reasoning [26]. There were suggestions that the form was an adjunct tool, rather than a tool that could cover reasoning across all aspects of the problem-solving process, as well as suggestions about how The Sensory Form could be enhanced to become a more comprehensive tool. The merits of The Sensory Form in terms of professional reasoning seem to suggest that it is best suited to being a tool that can be used getting therapists started along a path of reasoning that ensures links are made from the very beginning between sensory processing and participation.

Second, many therapists perceived that The Sensory Form users needed to have a level of knowledge and training in sensory processing in order to use the form effectively. Likert scale responses in relation to nonoccupational therapist use of The Sensory Form were divided, with half of the therapists reporting that they disagreed that parents would be able to use The Sensory Form. Parents play a key role in determining priorities for their children and family-centred practice is a key guiding philosophy in occupational therapy for children [46]. Incorporating parent perspectives, capacity building, and goals in an authentic way can lead to positive outcomes, as families are an important part of a child's team [38]. Findings from a study by Mora and Chapparo [47] indicated that parents can be trained and supported to implement sensory processing interventions. An identified strength of The Sensory Form was that it may provide a visual tool and platform to encourage collaboration with families and the team. However, one therapist reported that something simpler may be more appropriate for families. The Sensory Form may be an appropriate tool for therapists to codesign interventions with families to support sensory processing differences and may assist in the planning and reasoning process. However, given the current design of The Sensory Form, it may not be appropriate for parents to use without therapist support. Perceptions of parents were not included in the present study, and no studies have been conducted examining parents' perceptions of the suitability of The Sensory Form, or a modified parental version of the form. Parent perceptions about their own competence in the use of The Sensory Form may differ from therapists' views.

Similarly, therapists were also divided on their perceptions regarding teacher use of The Sensory Form with ten therapists disagreeing that it was suitable for teacher use. Therapist 2 raised concerns about use by professionals who do not necessarily understand the occupational perspective. Teachers are an important part of the school context [48] and are often expected to collaborate with occupational therapists in school settings [49]. Therapists identified that The Sensory Form may encourage collaboration between team members, which may have a positive outcome in a school setting, with teacher training and support. A study by Mills and Chapparo [50] found that sensory strategies could be accepted and utilised in a classroom setting, provided that teachers were included in the selection of sensory strategies, and were provided with training and mentoring in their use. Findings from studies facilitating teachers to use sensory strategies with training and support were positive [18]. To enable successful use of The Sensory Form by nonoccupational therapists such as parents and teachers, targeted training in the use of The Sensory Form would be necessary. Capturing the perceptions of teachers was out of scope for this study; however, teacher perceptions may have been different to those of therapists.

The third key finding is that changes may be required in order to enhance the usability of The Sensory Form. Sensory processing differences are a complex issue requiring individualised planning around the impact that these differences may have on a child's occupational performance [51]. Capturing an individual child's sensory processing patterns in an accurate way can be challenging, particularly when the child displays more than one sensory processing style [12], and when complex problems with participation are observed [6], which may or may not be sensory in nature [19]. There may be benefits associated with the presentation of complex information on one form, including giving therapists a visual guide to support their professional reasoning. Future research will involve a revised version of The Sensory Form which will be evaluated in terms of utility. The revised version of The Sensory Form will include more clarity around specific sensory processing issues and how these fit into Dunn's sensory processing framework, as well as more guidance around suitable intervention strategies.

### 4.1. Limitations

There are a number of limitations associated with this small study. While the 20 therapists who responded had substantial clinical experience supporting sensory processing differences, there may be perceptions not captured in this study that are relevant. Most of the occupational therapists who responded focussed their practice on supporting children. Authors believe that The Sensory Form may have application in supporting sensory processing differences across the lifespan; however, the utility of The Sensory Form for supporting sensory processing differences in an adult population remains largely unknown. The focus of this study was to gain the perceptions of occupational therapists as leading professionals in supporting sensory processing differences [8]. However, the perceptions of other health professionals, teachers, parents, and children themselves may have been different. Future research investigating The Sensory Form could involve the development and provision of training and mentoring to nonoccupational therapists in the use of The Sensory Form and further evaluating its utility with these users in supporting sensory processing differences. Further exploration of associated materials such as resources and worked examples may also be needed to ensure the utility of The Sensory Form as a tool that documents sound professional reasoning.

### 4.2. Implications for Practice

The Sensory Form may be a suitable tool to assist occupational therapists in their professional reasoning when addressing sensory processing differences as well as supporting collaboration with nonoccupational therapists. In particular, it provides a method for occupational therapists to record their observations and assessment of children's sensory processing differences within the context of daily activities and linking these observations directly with context-focused sensory interventions. This may be beneficial considering the lack of clarity in the literature regarding suitable interventions to assist children with sensory processing differences. The Sensory Form may be a useful tool, provided the user has knowledge and understanding of comprehensive sensory processing assessment within the context of occupation and participation. Further thought should be given to training and support of its use with those who are not occupational therapists, as well as occupational therapists with less experience.

## 5. Conclusions

This study was aimed at capturing the perceptions of occupational therapists about a one-page tool designed to assist with assessment and intervention planning in sensory processing practice. Occupational therapists reported strengths of The Sensory Form including its participation focus, utility, and capacity to enhance professional reasoning and collaboration. Therapists also reported weaknesses related to the need for background knowledge, limited support for professional reasoning, and poor clarity. Therapists also suggested changes to strengthen The Sensory Form including layout changes to enhance utility. Further research is needed to investigate the utility of a revised version of The Sensory Form and associated resources.

## Figures and Tables

**Table 1:** Therapist characteristics (*N* = 20).

Occupational therapy qualifications	Country of qualification	Years of occupational therapy practice experience	Years of practice experience with children	Practice area with children^∗^ (therapists could nominate more than one area)	Completed postgraduate training
Doctor of Philosophy (PhD) degree (*n* = 1), master's degree (*n* = 9), bachelor's degree (*n* = 10)	Australia (*n* = 15), UK (*n* = 4), South Africa (*n* = 1)	Mean (SD): 14.48 (11.87) Range: 1–40 Median: 10.5	Mean (SD): 11.7 (8.67) Range: 1–27 Median: 10	Schools (*n* = 12) Clinic (*n* = 8) Home visits (*n* = 6) Preschool (*n* = 2) CAMHS (*n* = 1) Hospital (*n* = 1) Adult clients in addition to children (*n* = 2)	Yes (*n* = 14) No (*n* = 6)

Key: SD: standard deviation; CAMHS: Child and Adolescent Mental Health Service. ^∗^Many therapists noted more than one practice area, so the “Practice area…” column adds to more than 20.

**Table 2:** Summary of therapist perspectives on The Sensory Form: strengths.

Participation focus (i) “The way that the form links body structures and functions with participation problems (occ. performance) in one form” (T11) (ii) “I really like that it takes into account participation” (T20) (iii) “the section on participation” (T18) (iv) “link between what is assessed/observed from sensory perspective back to participation” (T2)
Facilitates professional reasoning links between assessment and intervention (i) “I also like the way it leads the clinician to reflect on good autism practice as this is often the first step for intervention to support a greater degree of predictability in the environment that can lead to improved performance” (T11) (ii) “…acknowledges that there may be other strategies which can be used to support goals and behaviours which may be related to sensory processing” (T20) (iii) “I see it a clear and pragmatic way of expressing the complex principles of sensory processing and presenting it in a way which supports the intervention process, whether that is at home or school” (T8) (iv) “Structured and focused on goals and plans alongside the assessment data” (T7)
Encourages collaboration with others (i) “Also a good visual tool for when talking to non OT's about the child” (T4) (ii) “Could provide a good platform for and working through *[sensory processing issues]* with family and team” (T5) (iii) “It encourages collaboration with all team members” (T19)

Key: T: therapist participant.

**Table 3:** Summary of therapist perspectives on The Sensory Form: weaknesses.

Requires OT background knowledge, experience, and training (i) “Without specialist knowledge and training, developing strategies may be difficult” (T15) (ii) “I think… if you did not have a good understanding of SP *(sensory processing)* it may not be easy to use” (T20) (iii) “A knowledge of sensory processing areas is needed and this knowledge needs to be somewhat in-depth. I wonder how much training would be required to easily complete the form in an effective way” (T9) (iv) “I think it requires additional knowledge of sensory processing to ensure aspects aren't missed” (T6) (v) “Would be too hard for non OTs to use without specific training” (T12) (vi) “use by other professionals without understanding the form from an occupation perspective” (T2) (vii) “I think it would be great to develop something parents could use that is a simplified version of this” (T1)
Supports reasoning in a limited way (i) “May be difficult to measure progress using this form, I would need other assessments to be more thorough and inform intervention choices in addition to this form” (T5) (ii) “I see it as an adjunct to the use of standardised assessments such as the SP-2 (*Sensory Profile-2*); not as a stand-alone assessment, but rather a tool to support clinical reasoning and intervention planning” (T8) (iii) “…knowing how to interpret/analyse information appropriately” (T6)
Expand for clarity and logic (i) “I predict there may not be enough space in the boxes to add all relevant details, eg under logistics” (T19) (ii) “Visually confusing” (T13) (iii) “Could provide bigger boxes and become two sided. An ‘additional observations' section could be helpful” (T17)

Key: T: therapist participant.

## Data Availability

The qualitative survey data used to support the findings of this study are available from the corresponding author upon request.

## Supplementary Materials

Supplementary Materials Supplementary File One (S1): The Sensory Form webinar handout. This handout was given to participants to guide them through the use of The Sensory Form during the webinar.
