# Supplementary material for: A Survey of Occupational Therapists on a New Tool for Sensory Processing
Source: Occup Ther Int. 2020 Feb 28;2020:5909347. doi: 10.1155/2020/5909347 (PMC7068139; doi:10.1155/2020/5909347)
Supplement: Supplementary Materials — Supplementary File One (S1): The Sensory Form webinar handout. This handout was given to participants to guide them through the use of The Sensory Form during the webinar. [file 5909347.f1.pdf]

WESTERN SYDNEY
UNIVERSITY

WESTERN SYDNEY UNIVERSITY

The Sensory Form Webinar

Caroline Mills,
Occupational Therapist, Lecturer, Western Sydney University,

Background

WESTERN SYDNEY UNIVERSITY

- Sensory processing involves taking in a processing information from the senses (Bundy & Lane, 2012), an issue for typical children, ASD, ADHD (Dunn et al, 2016).
- Difficulties with sensory processing can negatively affect play, ADLs and participation (Dunn et al,2016) school participation for children with autism. (Ashburner et al, 2008).
- Disagreement in literature about the best approach (Bodison & Parham, 2018; Barton et al, 2015), little to guide practice (Case-Smith et al, 2015).
- Issues with defining intervention (Case-Smith, et al, 2015) and intervention fidelity (Schaaf et al, 2018).

What's the Plan?

1. Quick review- sensory processing

2. Meet "Tahlia"

3. Complete a sensory processing assessment and intervention plan for Tahlia using the sensory form

Sensory Processing-
"Taking in and organising sensory information for use"

Eight Senses - Outside senses- touch, taste, smell, hearing, vision

- Inside senses- Proprioception, vestibular, interoception

Why do we have senses?

Protection- *"Will it hurt me?"* (fight flight fright- protects the body from damage)

Discrimination- *"What is it?"* (more detailed information about objects and people

Habituation- "Do I need to keep paying attention?" (screening out irrelevant stimuli)

Winnie Dunn's model of sensory modulation (Dunn, 2014)

WESTERN SYDNEY UNIVERSITY

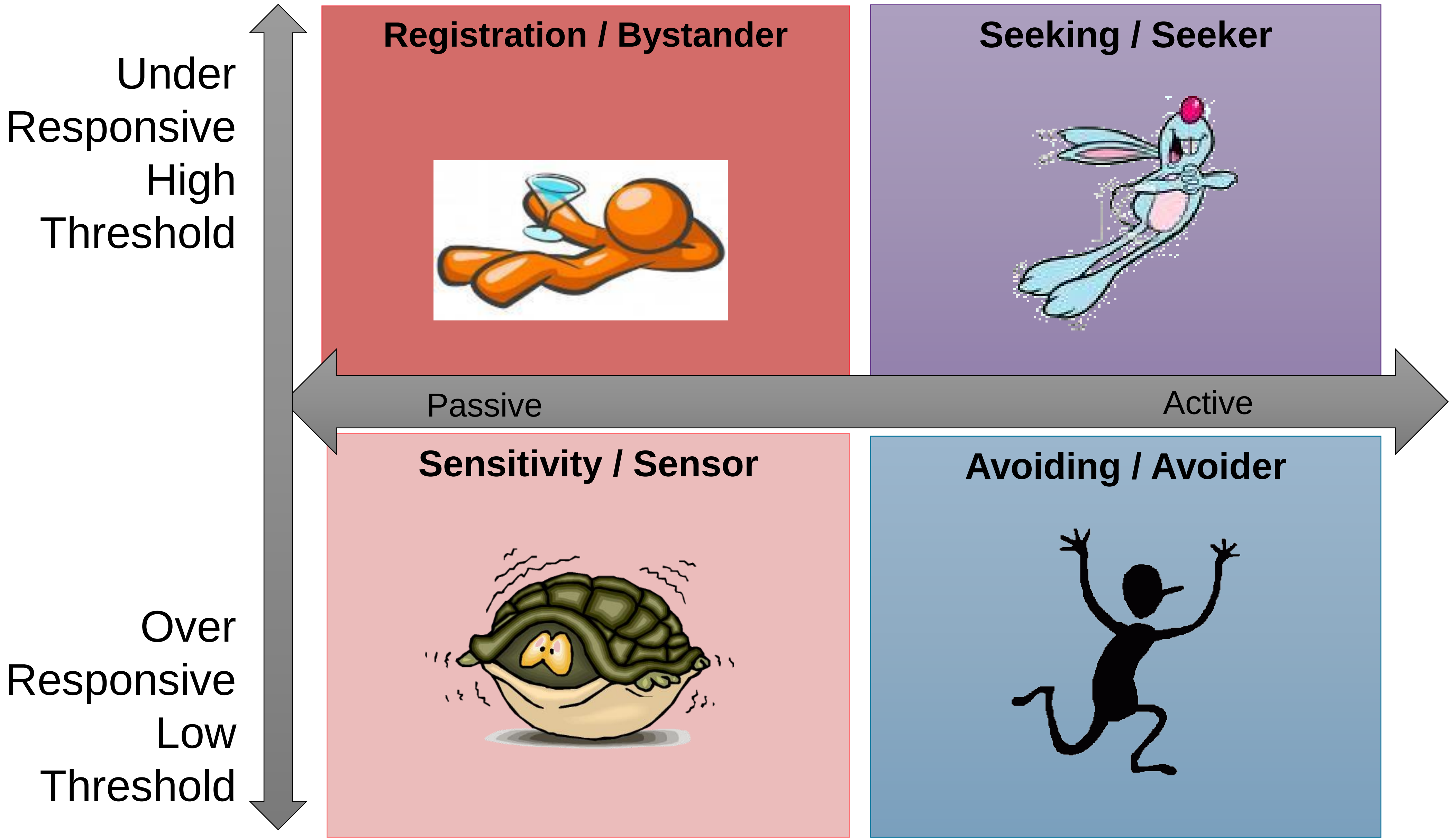

Sensory Processing- The Controversy

WESTERN SYDNEY UNIVERSITY

- Not enough evidence to warrant the use of sensory intervention (Barton et al, 2015)
- Should not be used for children with autism (Lang et al, 2012)
- Important to differentiate between Sensory Integration (fidelity measure- Parnham, et al, 2007) and Sensory Based Interventions (Case-Smith et al, 2015), Sensory Specific Techniques or Sensory Environmental Modification (Bodison & Parnham, 2018).
- Evidence is inconclusive (Watling & Hauer, 2015)
- Some evidence for SI with children with autism (Schaaf, Dumont, Arbesman & May-Benson, 2018)

If all you have is a hammer, everything looks like a nail.

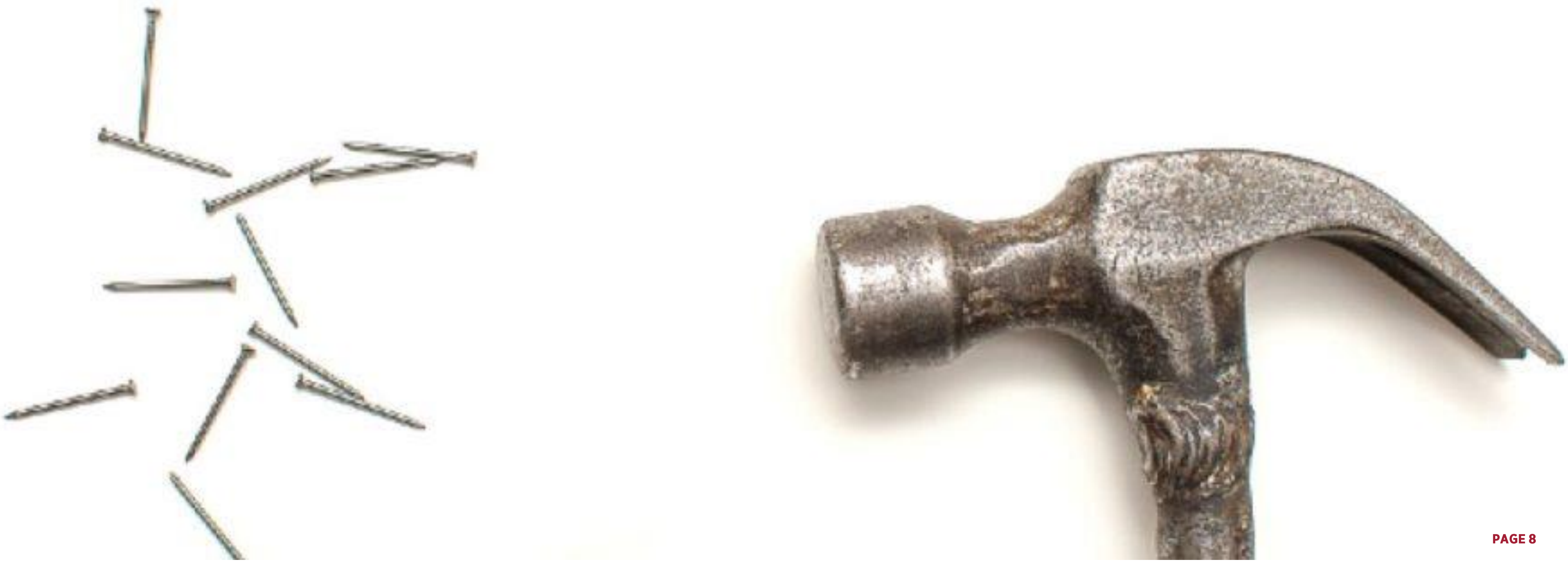

Providing Sensory Support

Universal Supports benefit all students:

- Structure
- Routine
- Predictability
- Information sharing
- Good Autism practice

Individualised Supports are needed for some students

- Gather information
- Understand sensory needs
- Provide specific sensory supports

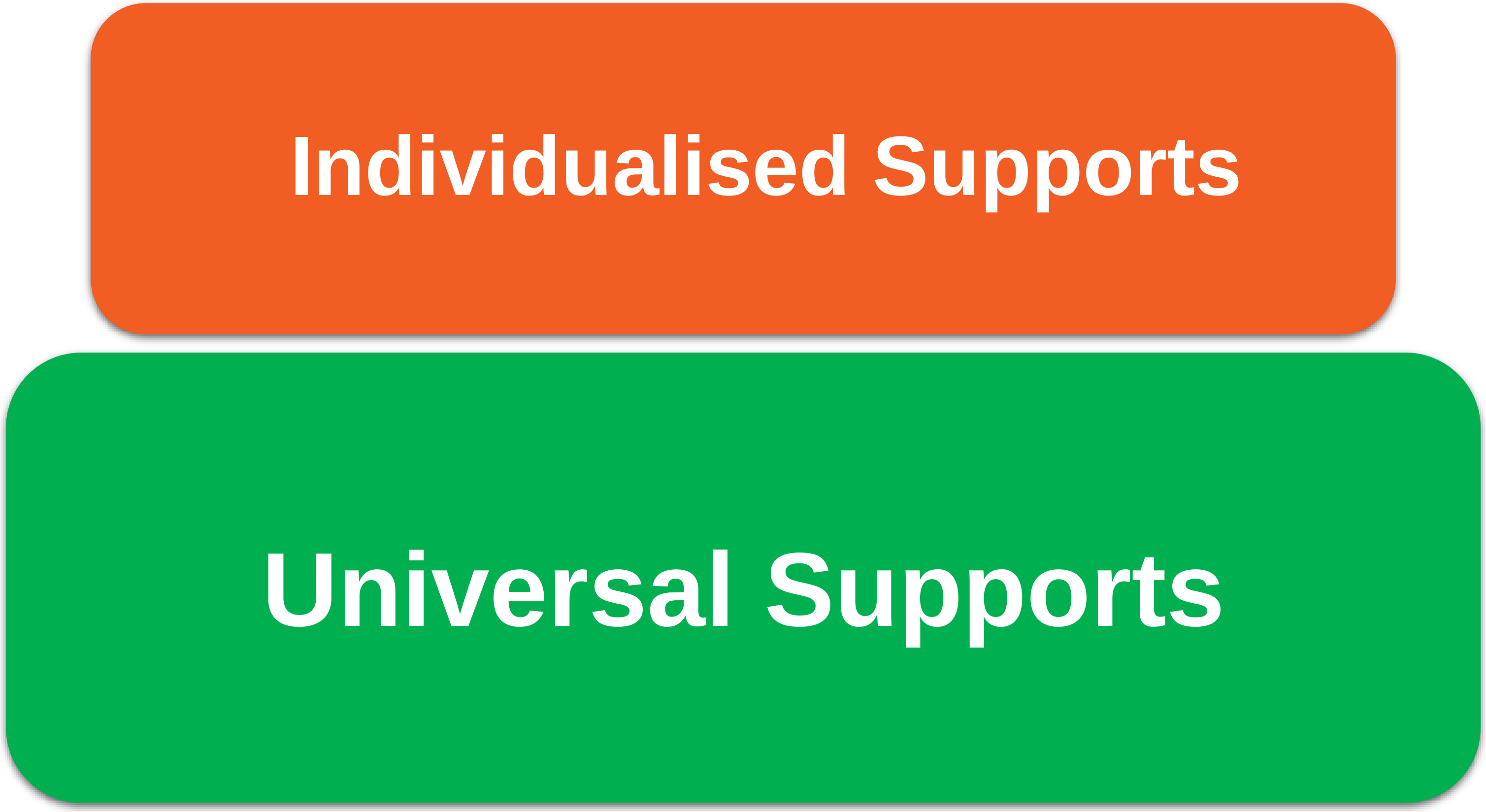

Sensory Processing Assessment and Intervention

WESTERN SYDNEY UNIVERSITY

- Do an assessment first! (AOTA, 2018)
- Then you can target strategies- do not use individual sensory interventions without assessment

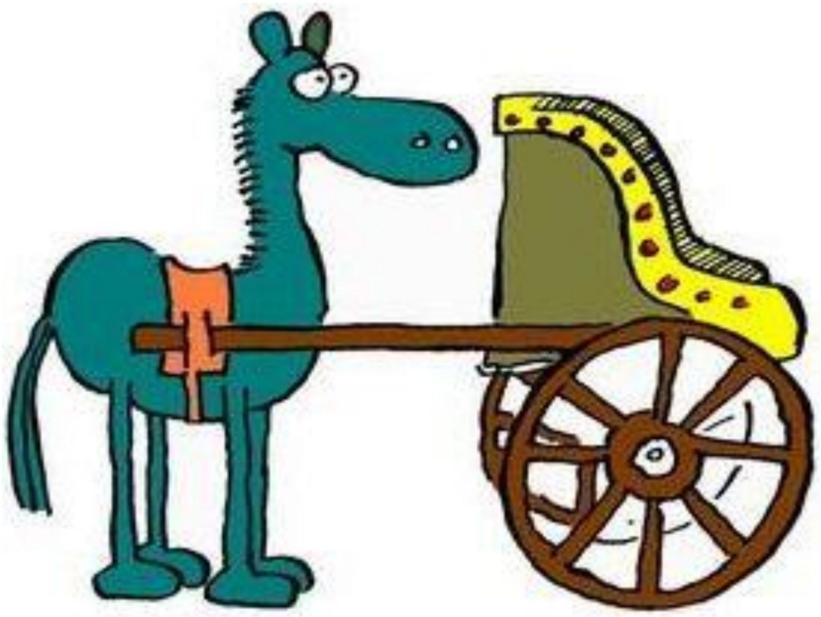

http://www.choosingwisely.org/aota-offers-choosing-wisely-list-of-practices-to-question/

autism spectrum AUSTRALIA

Name: Date: / / Who is in the team?

aspect practice

autism practice that works

VISION	SOUND	TOUCH	ORAL SENSORY	SMELL/TASTE	MOVEMENT (VESTIBULAR)	BODY (PROPRIOCEPTION)

Problems with Participation

Are you sure it's sensory? YES / NO If YES, approach with a sensory lens, If NO, alternative assessment or intervention

Bystander	Seeker	Avoider	Sensor

Is good autism practice in place? (structure, routine, predictability, visual supports etc.)

Proactive Strategies:

Environmental changes

Sensory activities

Teaching Coping Strategies:

Logistics:

Where, when, how??

Plan Review Date:

© Autism Spectrum Australia (Aspect)

WESTERN SYDNEY UNIVERSITY

The Sensory Form

You can access The Sensory Form for free here:

https://www.autismspectrum.org.au/content/sensory-processing-difficulties

Click on the Sensory Form box:

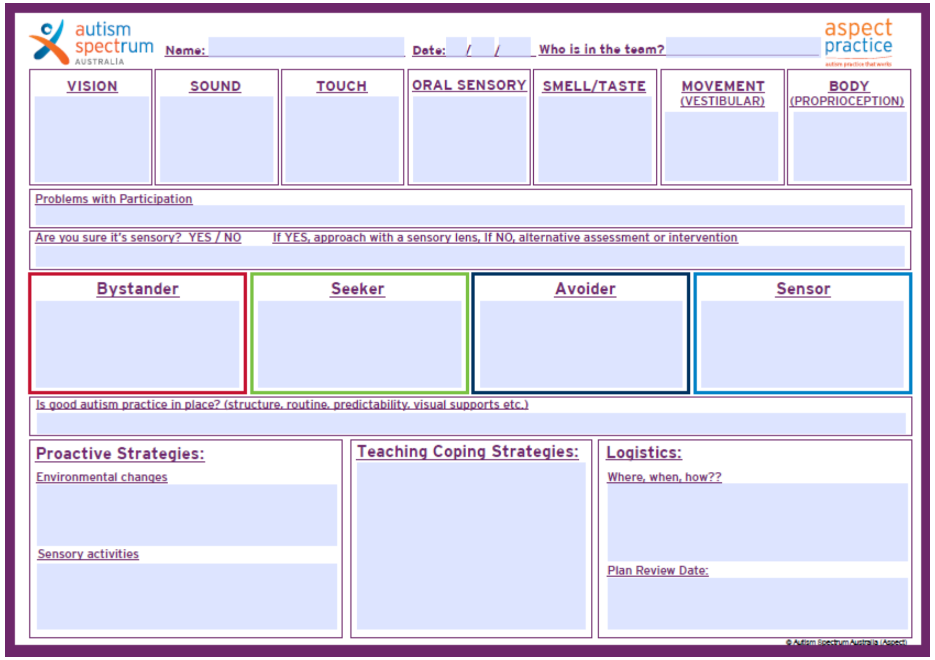

Let’s meet Thalia

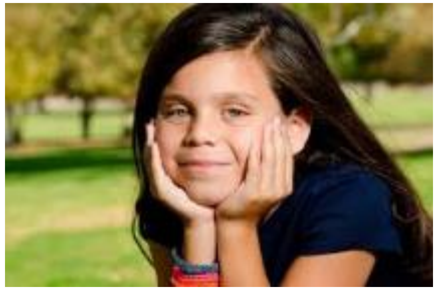

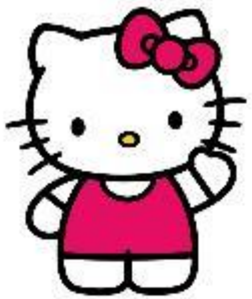

WESTERN SYDNEY UNIVERSITY

Thalia is a 10-year-old girl who enjoys listening to music and craft activities . She loves Hello Kitty and often draws Hello Kitty. Thalia has autism and an intellectual disability and attends a support class in a mainstream school.

Thalia’s mother has seen her dance to her favourite music when it comes on, but at other times she gets very upset by sound and puts her fingers over her ears. Her teacher at school has noticed this more than her mother at home.

Recently, the school have participated in an organised dance program with visiting dance instructors, but Thalia is reluctant to participate and hold hands with her partner. Thalia will go on a swing at the local park and seems to enjoy this, but is reluctant to participate in an organised soccer game at school. Her teacher reports that she’s quite clumsy, and becomes upset at times when she bumps into other people when moving around the school.

Thalia

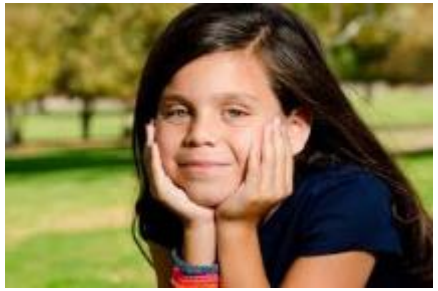

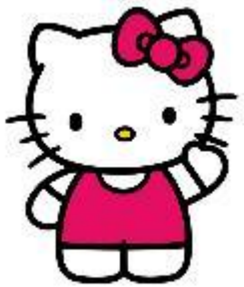

Thalia is an only child from a Greek Background who lives at home with her mother and her grandmother. Thalia lives in a three-bedroom house so Thalia has her own room. Thalia eats a variety of foods and enjoys cooking traditional Greek food with her grandmother.

Thalia will wear her school uniform but she complains to her mother that the tags cause itching and distract her at school sometimes. Thalia's mother says she likes to choose her own clothes.

Thalia

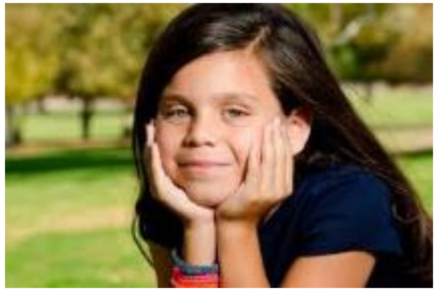

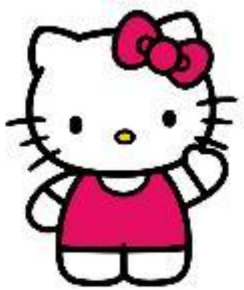

WESTERN SYDNEY UNIVERSITY

Imagine you are Thalia's occupational therapist. Recently you've noticed that she's having some difficulties at school. Her previous teachers and her mother have reported that Thalia is very different at home and school. She's in a smaller class with 15 students in total and she's having difficulties doing her independent work. Work time usually happens after playground time in the morning and after recess. Thalia says she can't concentrate with all the noise and she is worried about not being able to complete her work and then missing her 'fast finishers' craft activity. Her teacher has noticed that she fidgets with her hair and her pencil case and this affects her ability to complete the task within the time frame. Thalia has a visual schedule on her desk reminding her of the tasks she has to do. Thalia is able to communicate verbally with the teacher but she hasn't been doing this during work time.

Who's in the Team?

WESTERN SYDNEY UNIVERSITY

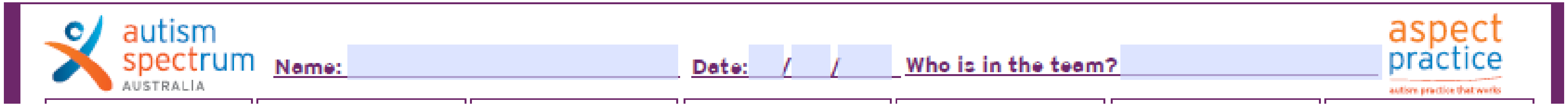
autism spectrum AUSTRALIA

Name: Date: / / Who is in the team?

aspect practice

- Important to consider the important people in the student's life.
- Insights from different environments- 'context' is very important (Ismael et al, 2018).
- Thalia, Mum, grandma, teacher.

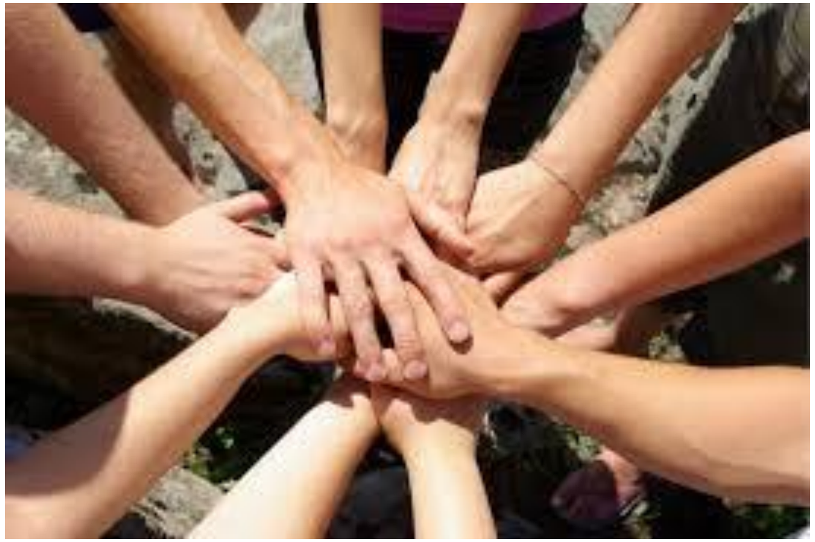

autism spectrum **Name:** **Date:** / / **Who is in the team?** aspect practice

VISION	SOUND	TOUCH	ORAL SENSORY	SMELL/TASTE	MOVEMENT (VESTIBULAR)	BODY (PROPRIOCEPTION)

Problems with Participation

Are you sure it's sensory? YES / NO If YES, approach with a sensory lens, If NO, alternative assessment or intervention

Bystander	Seeker	Avoider	Sensor

Is good autism practice in place? (structure, routine, predictability, visual supports etc.)

Proactive Strategies:

Environmental changes

Sensory activities

Teaching Coping Strategies:

Logistics:

Where, when, how??

Plan Review Date:

© Autism Spectrum Australia (Aspect)

WESTERN SYDNEY UNIVERSITY

The Sensory Form

Thalia’s Sensory Processing

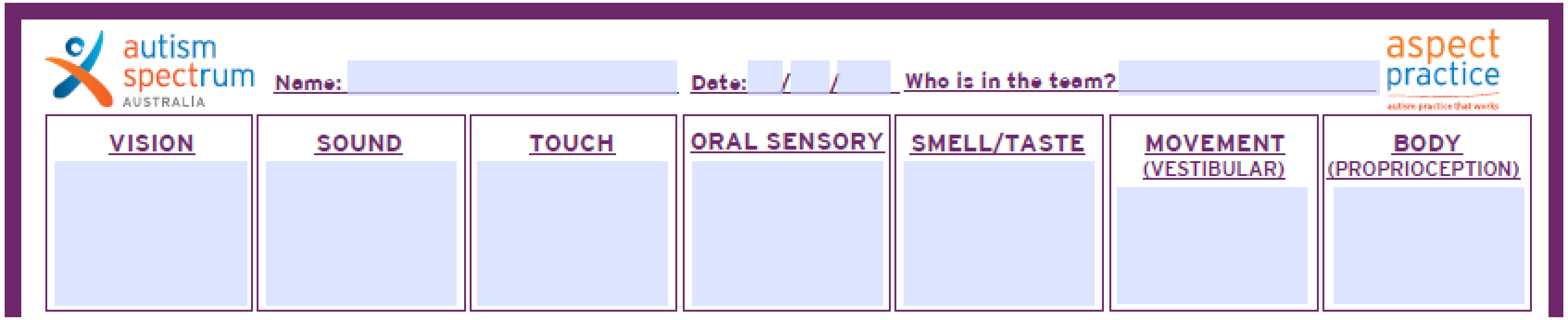
autism spectrum AUSTRALIA
Name: Date: / / Who is in the team?
aspect practice

VISION	SOUND	TOUCH	ORAL SENSORY	SMELL/TASTE	MOVEMENT (VESTIBULAR)	BODY (PROPRIOCEPTION)

Vision-
-Uses visual schedule

Hearing-
complains of noise
-Hands over ears
-Seeks choice of sound

Touch-
-fidgets
-issues with clothing
-issues with holding hands

Oral Sensory-Smell-Taste
- Strength
- Wide range of food
- Enjoys cooking

Movement
- Enjoys swing- doesn’t seek it out
- Dance- sometimes

Body (Proprioception)
- Clumsy- bumps into people during games

Thalia's Sensory Processing

WESTERN SYDNEY UNIVERSITY

Vision-	Hearing-	Touch-	Oral Sensory- Smell-Taste	Movement	Body (Proprioception)
-Uses visual schedule	complains of noise<br>-Hands over ears<br>-Seeks choice of sound	-fidgets<br>-issues with clothing<br>-issues with holding hands	- Strength<br>- Wide range of food<br>- Enjoys cooking	- Enjoys swing- doesn't seek it out<br>- Dance- sometimes	- Clumsy- bumps into people during games

Problems with Participation

Thalia's Problems with Participation:

Doing school work- touch and noise, fidgeting in class- does this help or hinder?

Difficulties after playground play, Different in different environments

"A problem isn't a problem until it's a problem"

Are you sure it’s sensory?

Are you sure it's sensory? YES / NO   If YES, approach with a sensory lens, If NO, alternative assessment or intervention

- Specific sensory observations and reports from Thalia- touch and noise
- There may be issues with a **“build up”** of sensory input- needing to focus on work after playground play
- Consider sensory aspects of the **playground**- noisy, unpredictable touch, social demands
- **Differences in different environments** suggests that Thalia is prone to notice a lot in the environment
- Differences observed when given **choice** and **control** of sensory input- eg. music and dance
- There is probably a **social** aspect to Thalia’s participation difficulties (not all sensory!)

autism spectrum AUSTRALIA

Name: **Date:** / / **Who is in the team?**

aspect practice
autism practice that works

VISION	SOUND	TOUCH	ORAL SENSORY	SMELL/TASTE	MOVEMENT (VESTIBULAR)	BODY (PROPRIOCEPTION)

Problems with Participation

Are you sure it's sensory? YES / NO   If YES, approach with a sensory lens, If NO, alternative assessment or intervention

Bystander	Seeker	Avoider	Sensor

Is good autism practice in place? (structure, routine, predictability, visual supports etc.)

Proactive Strategies:

Environmental changes

Sensory activities

Teaching Coping Strategies:

Logistics:

Where, when, how??

Plan Review Date:

© Autism Spectrum Australia (Aspect)

WESTERN SYDNEY UNIVERSITY

What type of sensory style does Thalia have?

Winnie Dunn's model of sensory modulation (Dunn, 2014)

WESTERN SYDNEY UNIVERSITY

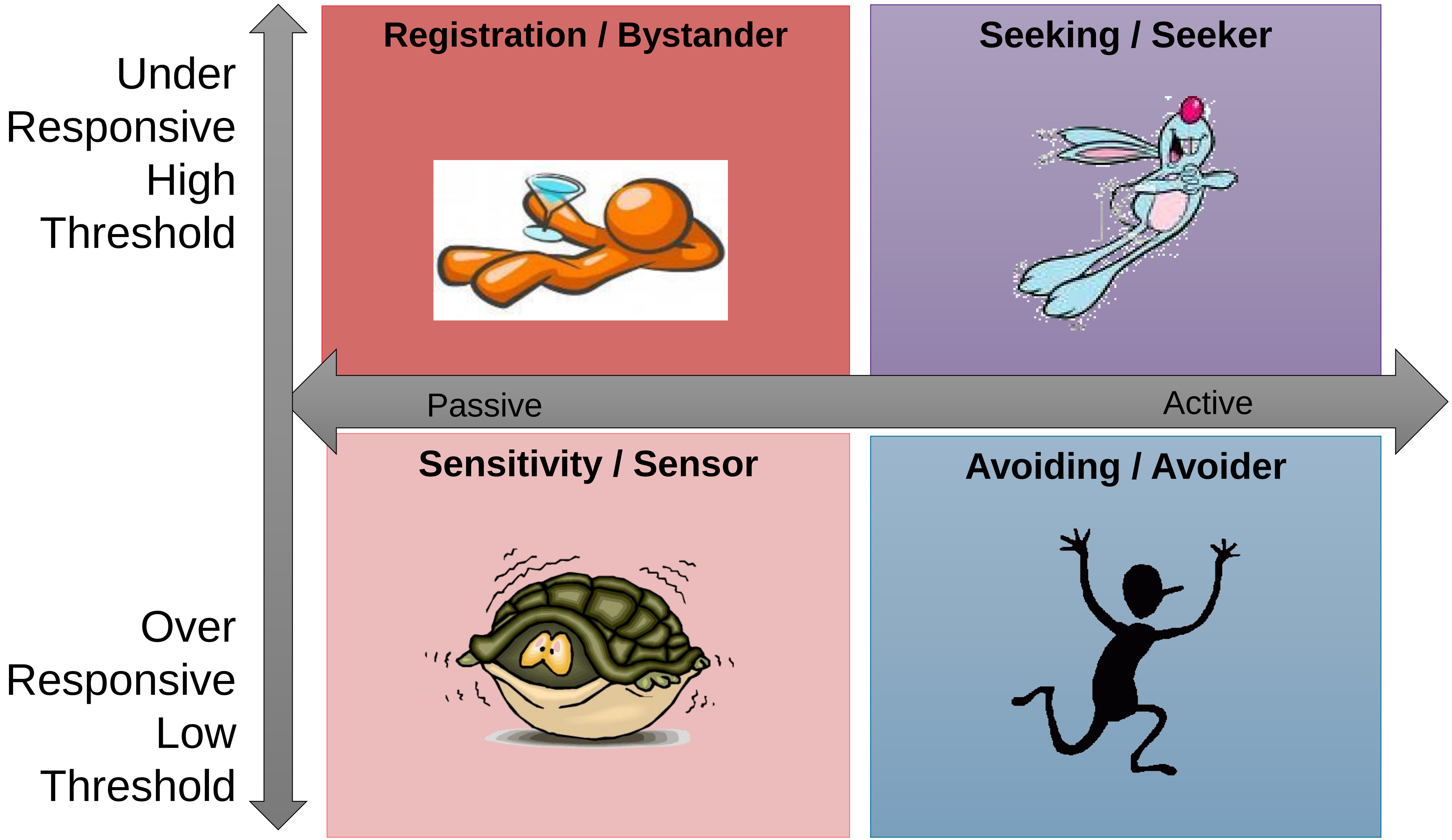

Winnie's Dunn's model- Four Sensory Styles

- People don't fit neatly into these categories!
- Children can fit into more than one style.
- Thalia- Avoider
- Distress- noise, noisy environments, avoids activities
- Seeker- fidgeting-
- Coping mechanism?
- Active Sensory styles

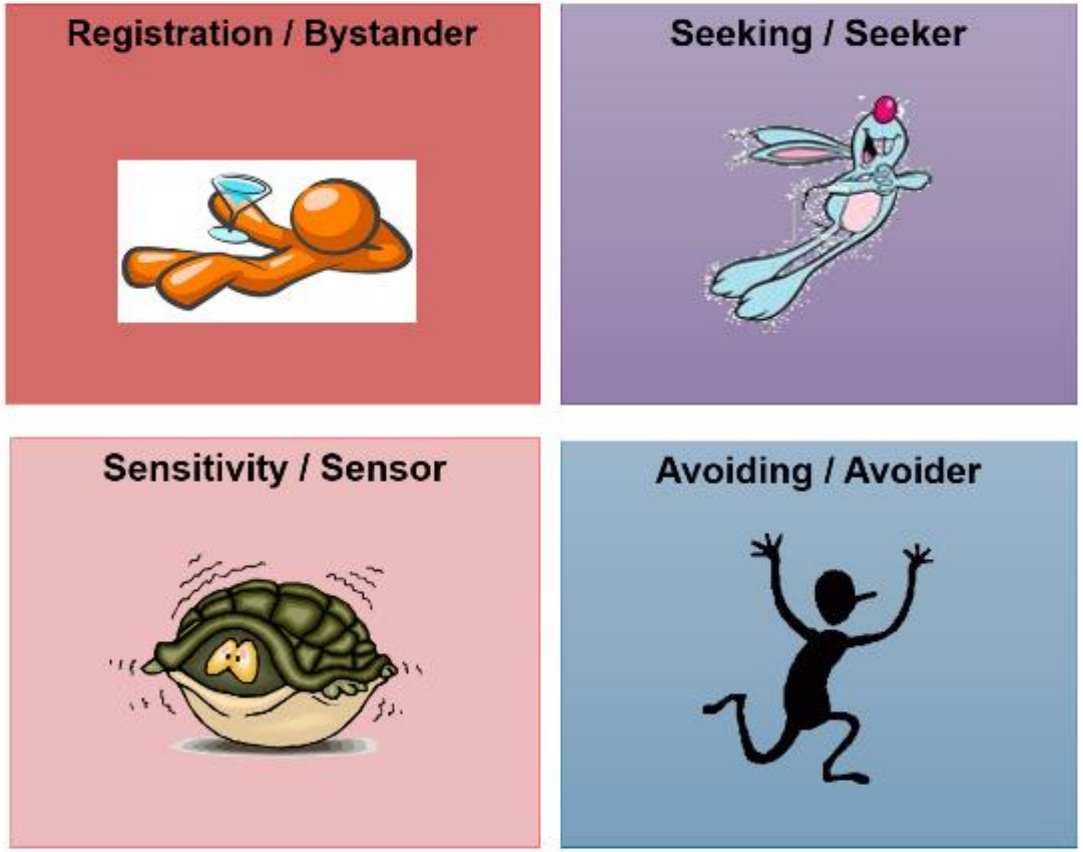

autism spectrum AUSTRALIA

Name: **Date:** / / **Who is in the team?**

aspect practice

VISION	SOUND	TOUCH	ORAL SENSORY	SMELL/TASTE	MOVEMENT (VESTIBULAR)	BODY (PROPRIOCEPTION)

Problems with Participation

Are you sure it's sensory? YES / NO If YES, approach with a sensory lens, If NO, alternative assessment or intervention

Bystander	Seeker	Avoider	Sensor

Is good autism practice in place? (structure, routine, predictability, visual supports etc.)

Proactive Strategies:

Environmental changes

Sensory activities

Teaching Coping Strategies:

Logistics:

Where, when, how??

Plan Review Date:

© Autism Spectrum Australia (Aspect)

WESTERN SYDNEY UNIVERSITY

Complete this section with Thalia's sensory style

☆ You don't need to fill in every box

Good Autism Practice

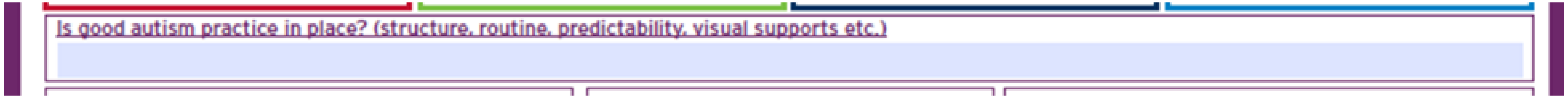
Is good autism practice in place? (structure, routine, predictability, visual supports etc.)

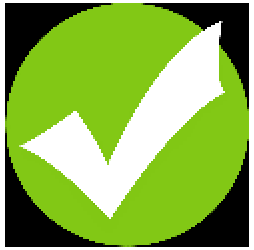

What is currently in place?

- Visual Schedule
- Routine
- Is this meeting Thalia’s needs?

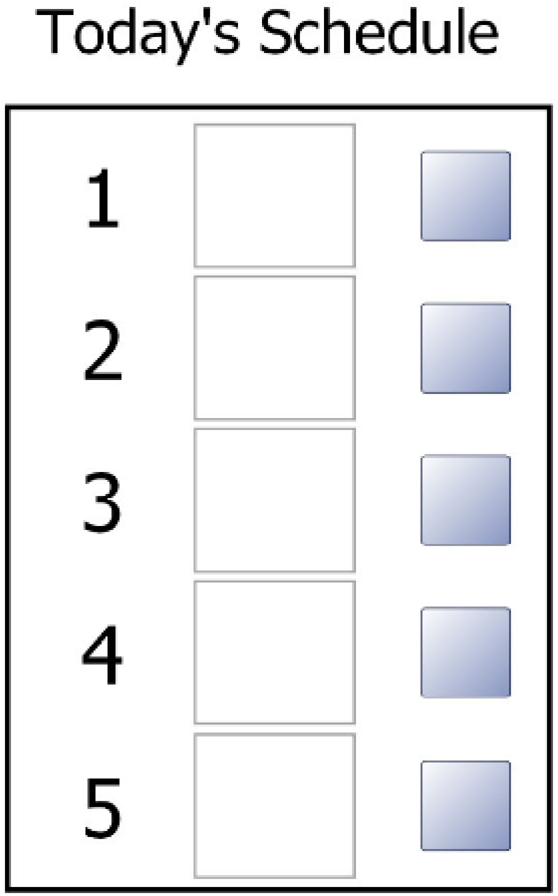

The Bottom of The Sensory Form: Intervention

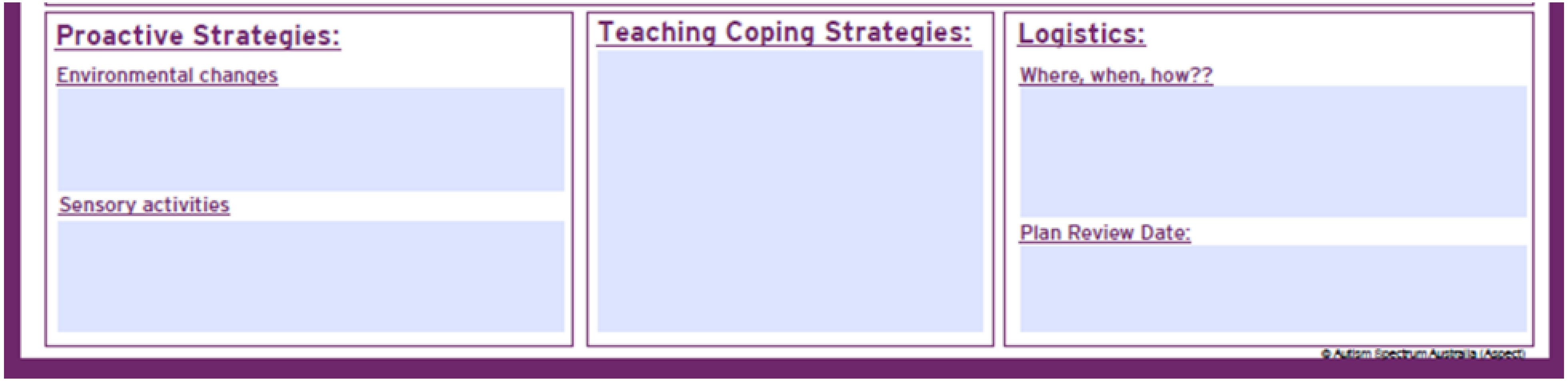

Proactive Strategies:	Teaching Coping Strategies:	Logistics:
Environmental changes		Where, when, how??
Sensory activities		Plan Review Date:

© Autism Spectrum Australia (Aspect)

Intervention Strategies matched to Thalia's needs.

- Environmental Changes- help or hinder participation
- Sensory Activities- safe and appropriate way to access sensory input
- Coping Strategies- What strategies can we teach Thalia to help her?
- Logisitics- How are we going to pull it all together?

Environmental Changes

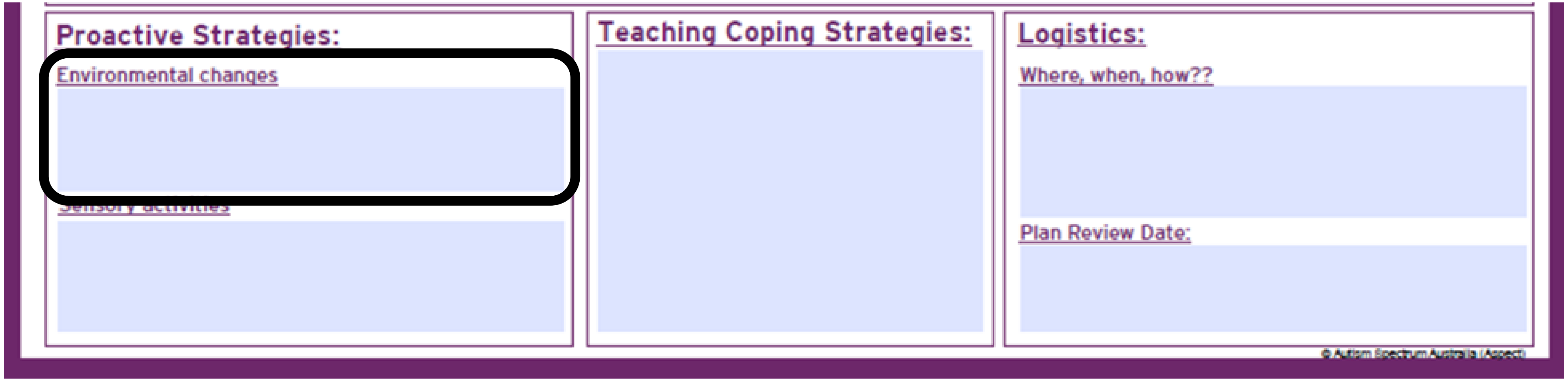

- Break space- take a break from sensory input
- Structure this- break cards (3 breaks per session)
- Headphones

Sensory Activities

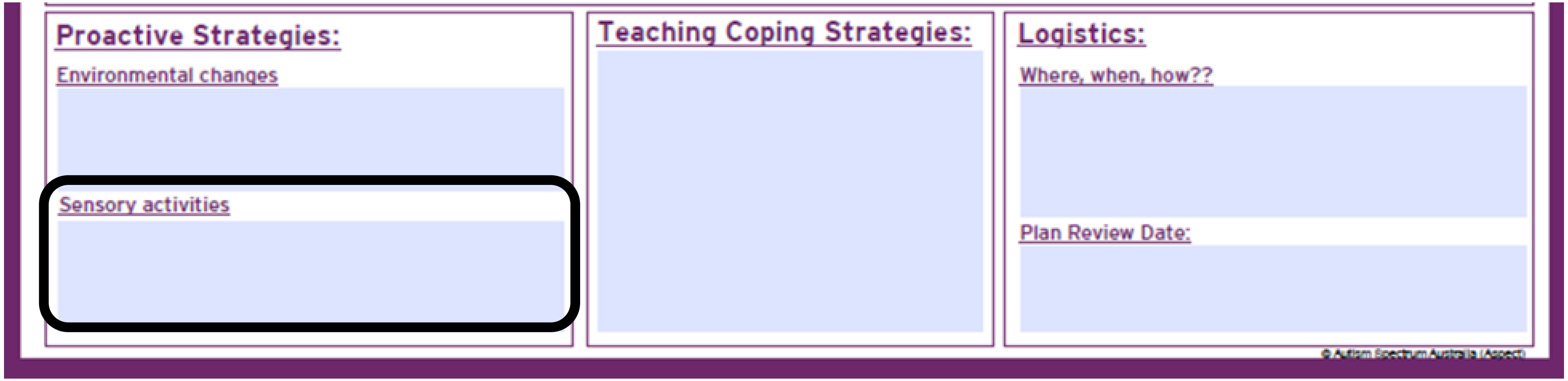

Proactive Strategies:

Environmental changes

Sensory activities

Teaching Coping Strategies:

Logistics:

Where, when, how??

Plan Review Date:

© Autism Spectrum Australia (Aspect)

- Fidget toys- trial of different toys to see which work best for Thalia and for the environment

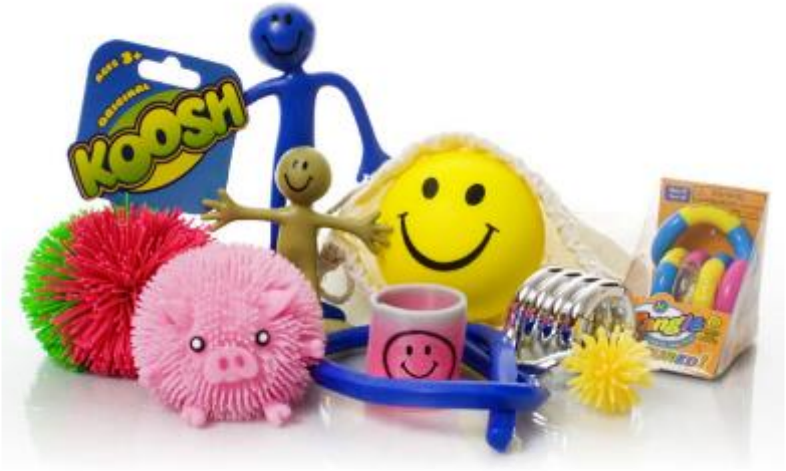

Teaching Coping Strategies

WESTERN SYDNEY UNIVERSITY

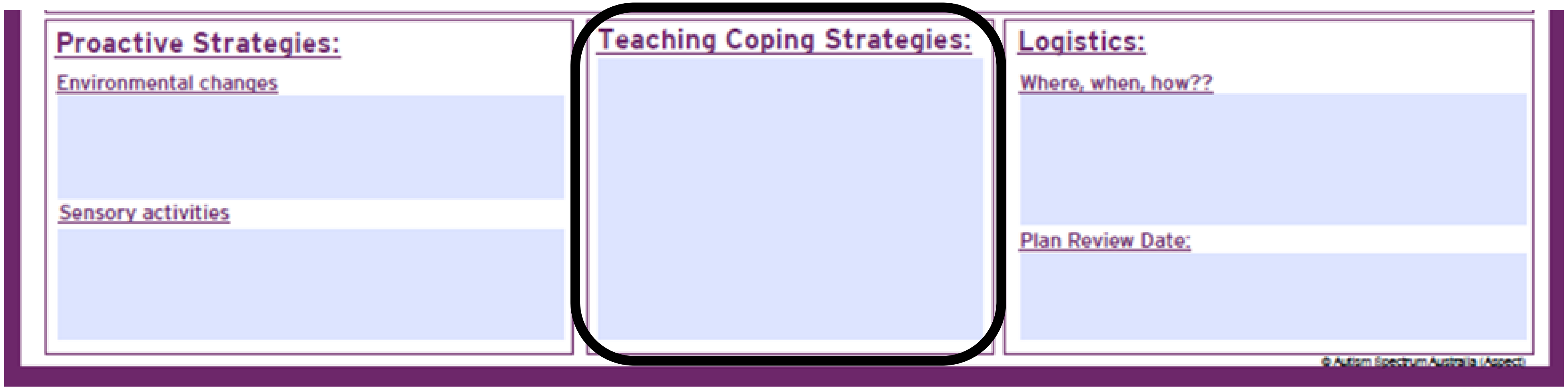

- Feelings chart/thermometer- *How am I feeling? What can I do when it's all too much…?*
- Social/Sensory Story- *When the environment is too noisy I can…*

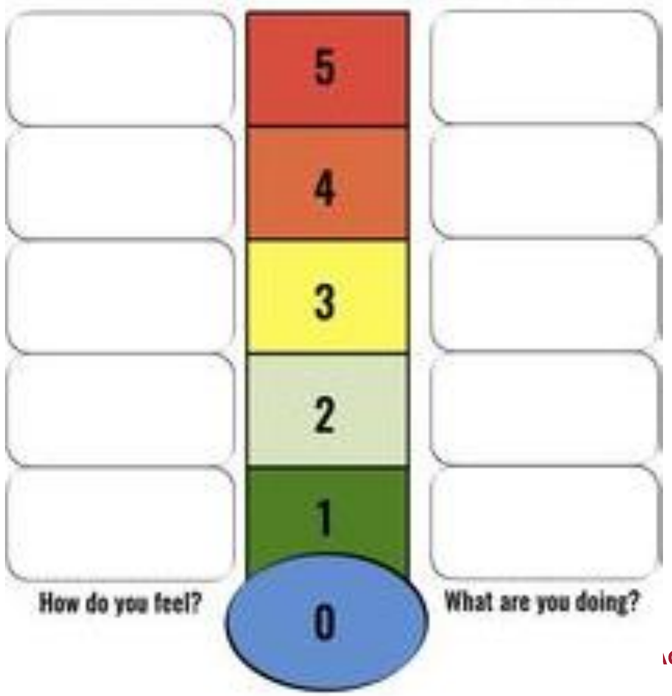

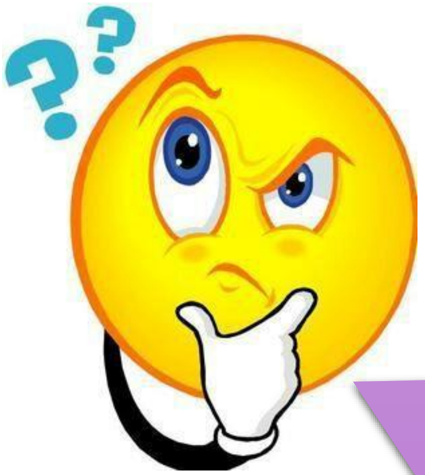

But those strategies aren't sensory??

- ✓ It's ok to address sensory issues using non-sensory strategies!
- ✓ The aim is to increase **participation**

Logistics: Where, When, How?

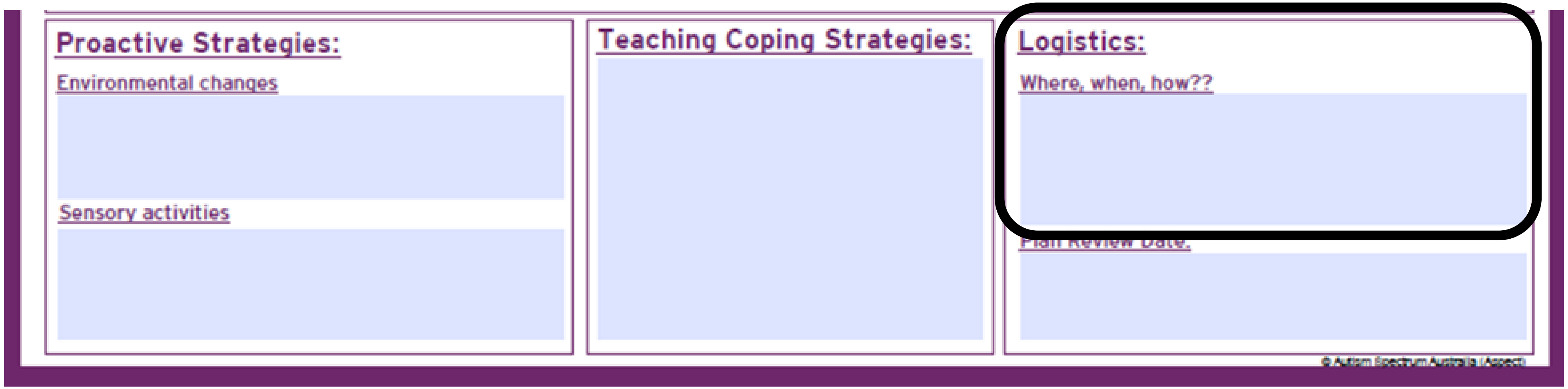

- School and home- available space- Breakout space
- Team meeting- allocate tasks:
- What to buy- Fidget toys, headphones
- Creating thermometer, writing social story, making break cards
- Get Thalia's input and buy in

Logistics- Make a Plan- The Checklist

WESTERN SYDNEY UNIVERSITY

Tasks	Who?	When?
Meet with home and school		
Review classroom and school supports		
Scope out breakout space		
Shop for fidget toys to trial		
Buy Headphones		
Create thermometer		
Create Break cards		
Write social story		

Reviewing the Plan

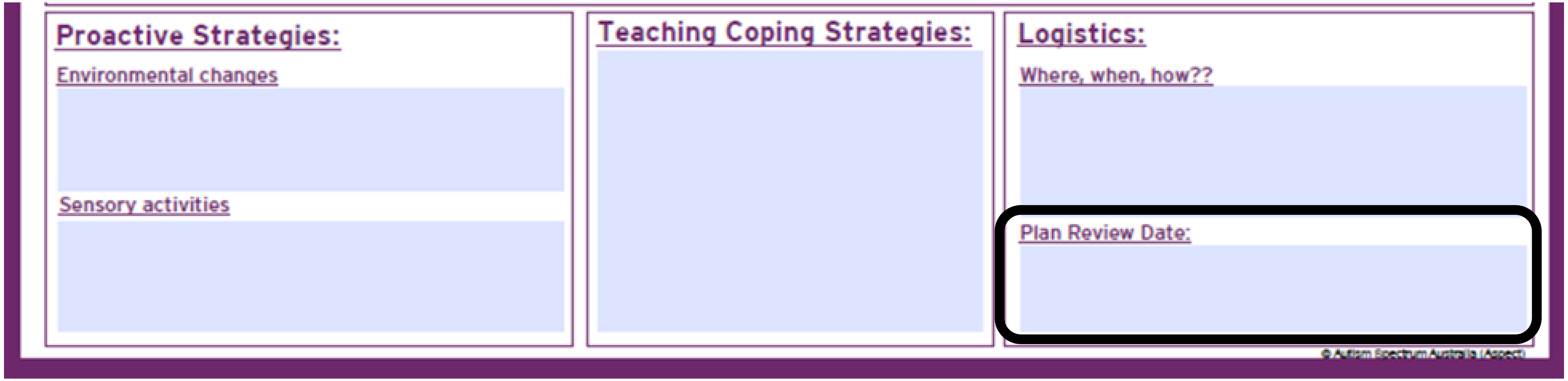

- Important to review your plan!
- 4-8 weeks
- Go back to 'Problems with Participation'
- What's the goal?
- Is it time to call in an OT?

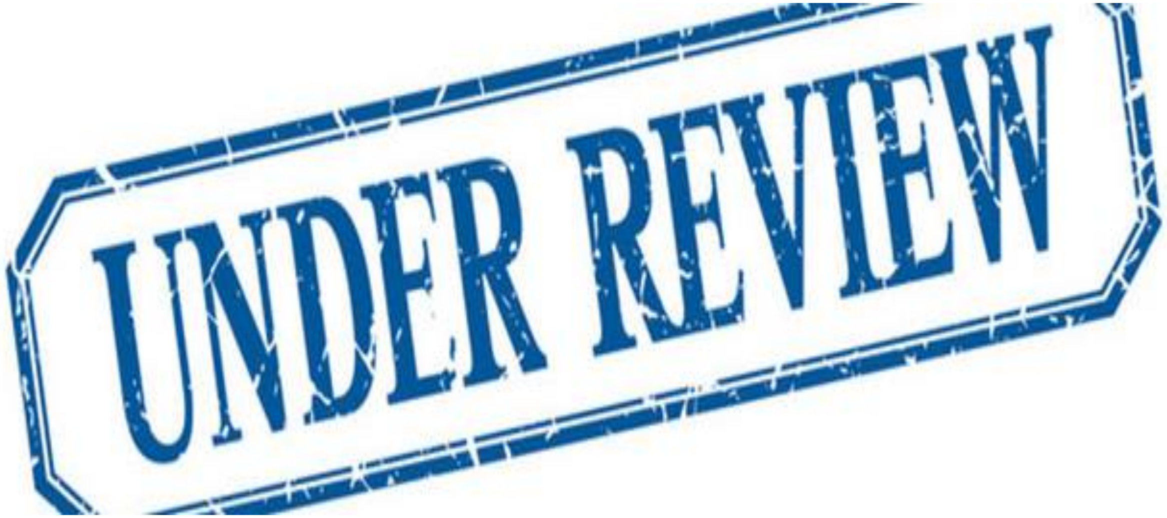

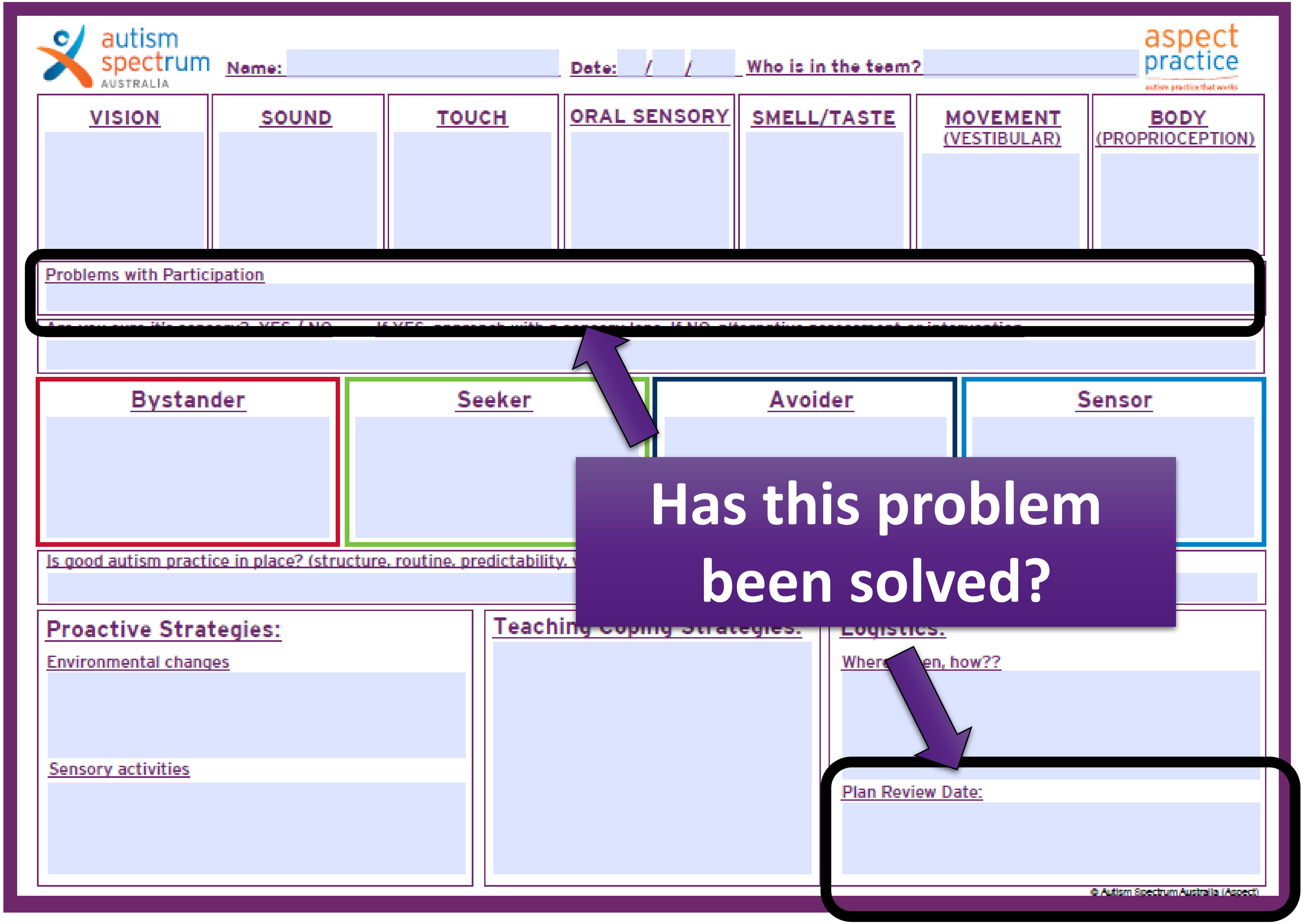

WESTERN SYDNEY UNIVERSITY

Plan Review

Conclusion

WESTERN SYDNEY UNIVERSITY

- You can work through the process of solving sensory problems to assist a person with their occupational performance and participation.

- There are no quick answers, it's a problem solving process!

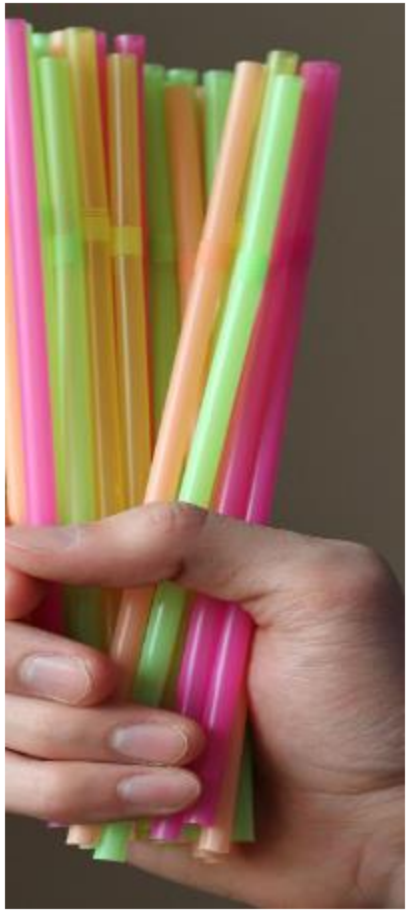

The Sensory Form is Free Online!

WESTERN SYDNEY UNIVERSITY

https://www.autismspectrum.org.au/content/sensory-processing-difficulties

Click on the Sensory Form box:

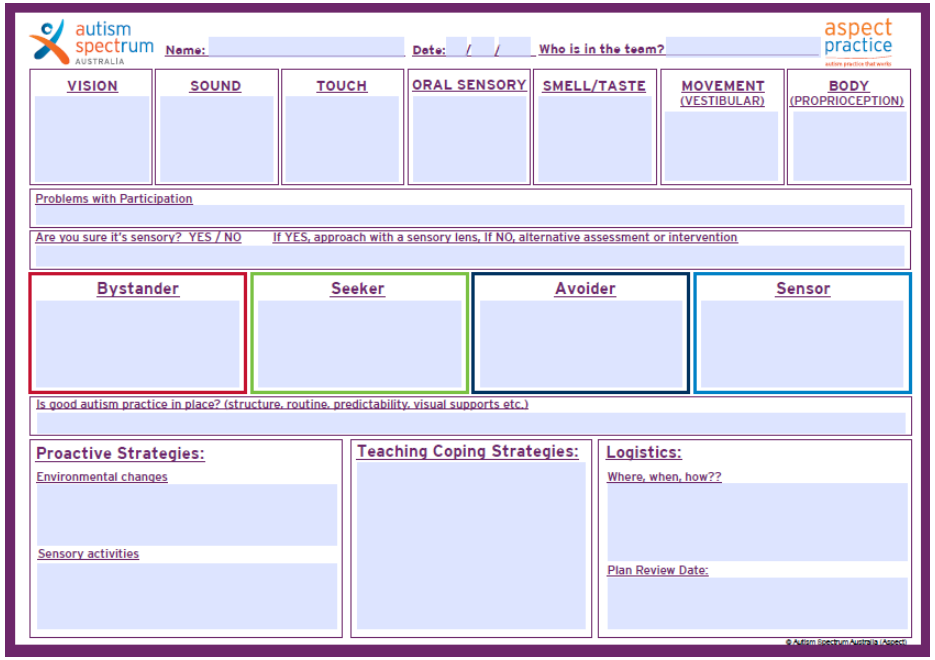

autism spectrum AUSTRALIA

WESTERN SYDNEY
UNIVERSITY

Caroline Mills
Occupational Therapist, Lecturer, Western Sydney University
Caroline.mills@westernsydney.edu.au

References

WESTERN SYDNEY UNIVERSITY

Ashburner, J., Ziviani, J., & Rodger, S. (2008). Sensory processing and classroom emotional, behavioral, and educational outcomes in children with autism spectrum disorder. *American Journal of Occupational Therapy, 62,* 564–573.

Barton, E.E., Reichow, B., Schnitz, A., Smith, I.C. & Sherlock, D. (2015). Review article: A systematic review of sensory-based treatments for children with disabilities *Research in Developmental Disabilities 37,* 64–80

Bodison, S. C., & Parham, L. D. (2018). Specific sensory techniques and sensory environmental modifications for children and youth with sensory integration difficulties: A systematic review. *American Journal of Occupational Therapy, 72*, 7201190040. https://doi.org/10.5014/ajot.2018.029413

Case-Smith, J., Weaver, L.L. & Fristad, M.A. (2015). A systematic review of sensory processing interventions for children with autism spectrum disorders. *Autism* 19(2), 133-48.

Dunn, W. (2014). *Sensory Profile 2*. Bloomington, MN: Pearson Psychcorp.

Dunn, W., Little, L., Dean, E., Robertson, S. & Evans, B. (2016). The state of the science on sensory factors and their impact on daily life for children: A scoping review. Occupation, Participation and Health, 36 (2S), 3S-26S.

Ismael, N., Lawson, L. M., & Hartwell, J. (2018). Relationship between sensory processing and participation in daily occupations for children with autism spectrum disorder: A systematic review of studies that used Dunn's sensory processing framework. American Journal of Occupational Therapy, 72, 7203205030. https://doi.org/10.5014/ajot.2018.024075

References

WESTERN SYDNEY UNIVERSITY

Lecavalier L, Leone S,& Wiltz J. (2006). The impact of behaviour problems on caregiver stress in young people with autism spectrum disorders. *Journal of Intellectual Disability Research 50*(3), 172-183.

Lang, R., O'Reilly, M., Healy, O., Rispoli, M., Lydon, H. et al (2012). Sensory integration therapy for autism spectrum disorders: A systematic review. *Research in Autism Spectrum Disorders 6*, 1004-1018.

Little, L.M., Dean, E. Tomchek, S.D. & Dunn, W. (2017): Sensory Processing Patterns in Autism, Attention Deficit Hyperactivity Disorder, and Typical Development, *Physical & Occupational Therapy In Pediatrics*, DOI: 10.1080/01942638.2017.1390809

Parham, L. D., Cohn, E. S., Spitzer, S., Koomar, J. A., Miller, L. J., Burke, J. P., et al. (2007). Fidelity in sensory integration intervention research. *American Journal of Occupational Therapy, 61*, 216–227.

Schaaf, R. C., Dumont, R. L., Arbesman, M., & May-Benson, T. A. (2018). Efficacy of occupational therapy using Ayres Sensory Integration®: A systematic review. American Journal of Occupational Therapy, 72, 7201190010. https://doi. org/10.5014/ajot.2018.028431Issues

Syriopoulou-Delli, C.K., Cassimos, D.C., Tripsianis, G.I. & Polychronopoulou, S.A. (2012). Teachers' perceptions regarding the management of children with autism spectrum disorders. *Journal of Autism and Developmental Disorders, 42*, 755–768.

Watling, R., & Hauer, S. (2015). Effectiveness of Ayres Sensory Integration® and sensory-based interventions for people with autism spectrum disorder: A systematic review. American Journal of Occupational Therapy, 69, 6905180030. http://dx.doi.org/10.5014/ajot.2015.018051
